# Peripheral Giant Cell Granuloma Mimicking Central Giant Cell Granuloma in a Pediatric Patient: A Diagnostic Challenge

**DOI:** 10.1155/crid/8935257

**Published:** 2026-09-12

**Authors:** Dante Alejandro Pineda Noceda, Raúl Mendoza García, Yamely Ruiz Vázquez, María Elena Llarena del Rosario

**Affiliations:** ^1^ Department of Pediatric Stomatology, National Institute of Pediatrics, Mexico City, Mexico, pediatria.gob.mx; ^2^ Faculty of Dentistry, National Autonomous University of Mexico (UNAM), Mexico City, Mexico, unam.mx

**Keywords:** child, giant cells, gingival diseases, peripheral giant cell granuloma

## Abstract

**Introduction:**

Peripheral giant cell granuloma (PGCG) is a benign reactive lesion that typically arises from the gingiva or edentulous alveolar ridge. Although superficial pressure resorption of the underlying bone has been described, extensive cortical erosion mimicking a central giant cell granuloma (CGCG) is exceptionally uncommon, particularly in pediatric patients, creating a significant diagnostic challenge.

**Case Presentation:**

A 10‐year‐old boy presented with an exophytic gingival mass involving the right mandibular alveolar ridge. Although the lesion appeared clinically peripheral, cone‐beam computed tomography demonstrated a 40 × 25 mm expansile osteolytic lesion with cortical bone erosion, involvement of adjacent developing teeth, and imaging findings suggestive of CGCG. Incisional biopsy revealed features consistent with PGCG. Laboratory investigations, including serum calcium, phosphorus, alkaline phosphatase, and parathyroid hormone levels, were within normal limits, excluding hyperparathyroidism. Complete surgical excision with peripheral osteotomy was performed under general anesthesia, and histopathological examination of the excised specimen confirmed the diagnosis of PGCG.

**Conclusion:**

This case illustrates that PGCG may rarely present with cortical bone erosion and radiographic findings closely resembling CGCG. Accurate diagnosis requires integration of clinical examination, advanced imaging, histopathological evaluation, and laboratory investigations rather than reliance on a single diagnostic modality. Recognition of this uncommon presentation may prevent misdiagnosis and avoid unnecessarily aggressive treatment in pediatric patients.

## 1. Introduction

Peripheral giant cell granuloma (PGCG) is a benign, reactive, nonneoplastic lesion that develops exclusively on the gingiva or edentulous alveolar ridge. It is believed to originate from the periosteum or periodontal ligament as a response to chronic local irritation or trauma, including dental plaque, calculus, food impaction, defective restorations, tooth extraction, periodontal disease, and other inflammatory stimuli. Although its precise pathogenesis remains uncertain, current evidence supports an exaggerated reparative response mediated by osteoclast‐like multinucleated giant cells within a fibrovascular stroma. PGCG represents one of the most common reactive giant cell lesions of the oral cavity and should be distinguished from other benign and malignant gingival proliferations because of its distinct biological behavior and therapeutic implications. [1–5]

Clinically, PGCG usually appears as a solitary sessile or pedunculated nodular mass with a smooth or ulcerated surface and a characteristic red, purple, or bluish coloration caused by its rich vascularity and repeated hemorrhage. Most lesions measure less than 2 cm in diameter and are asymptomatic, although larger lesions may interfere with mastication, speech, oral hygiene, or esthetics. PGCG occurs predominantly in the mandibular gingiva, particularly anterior to the first molars, and most frequently affects adults during the fourth to sixth decades of life, with a slight female predominance. Pediatric cases are uncommon, representing only a small proportion of reported cases, and therefore remain an important source of clinical information regarding diagnosis and management in growing patients. [1, 3, 6, 7]

Histopathologically, PGCG is characterized by numerous multinucleated giant cells dispersed within a highly cellular fibrovascular connective tissue stroma containing spindle‐shaped mesenchymal cells, abundant capillaries, hemorrhage, hemosiderin deposits, chronic inflammatory infiltrate, and occasionally reactive osteoid or immature bone formation. However, these microscopic characteristics are not pathognomonic and may overlap considerably with other giant cell lesions of the jaws. Consequently, histopathological examination alone is insufficient to establish the diagnosis, emphasizing the need for careful correlation with clinical presentation and radiographic findings. [3, 5, 8]

The differential diagnosis of PGCG includes several reactive and neoplastic gingival lesions, such as pyogenic granuloma, peripheral ossifying fibroma, peripheral odontogenic fibroma, hemangioma, metastatic lesions, and Brown tumor associated with hyperparathyroidism. Among these entities, central giant cell granuloma (CGCG) deserves particular attention because both lesions share remarkably similar histopathological features despite their distinct biological behavior, anatomical origin, treatment strategies, and prognosis. Failure to recognize these differences may lead to inappropriate surgical planning or unnecessary aggressive treatment. [3, 4, 8, 9]

CGCG is a benign intraosseous lesion that primarily affects children and young adults and exhibits a highly variable biological behavior ranging from indolent lesions to rapidly growing aggressive forms. Aggressive CGCGs frequently present with cortical expansion or perforation, root resorption, tooth displacement, pain, and increased recurrence rates. Radiographically, they usually appear as uni‐ or multilocular radiolucencies, often crossing the midline in the anterior mandible. Because of their locally destructive potential, management may require extensive curettage, peripheral ostectomy, en bloc resection, or adjunctive pharmacological therapies including corticosteroids, calcitonin, interferon‐*α*, or denosumab in selected cases. Recent multicenter studies have emphasized that treatment should be individualized according to the lesion′s biological behavior while balancing recurrence risk against surgical morbidity. [8, 10–13]

Although PGCG is classically described as an extraosseous lesion, superficial pressure resorption of the underlying alveolar bone has occasionally been reported. In exceptional circumstances, cortical erosion may become sufficiently pronounced to mimic an intraosseous giant cell lesion radiographically, creating a challenging diagnostic dilemma. Because both PGCG and CGCG contain virtually identical multinucleated giant cells histologically, the distinction between them cannot rely solely on microscopic evaluation. Instead, accurate diagnosis requires integration of clinical findings, radiographic characteristics, histopathological analysis, and biochemical investigations to exclude systemic conditions such as hyperparathyroidism that may present with giant cell lesions of the jaws. [2–4, 9, 12, 14]

The therapeutic approach for PGCG consists of complete surgical excision extending to the periosteum or periodontal ligament, accompanied by elimination of all local irritative factors to minimize recurrence. Reported recurrence rates vary between approximately 5% and 20%, with incomplete excision, persistence of local irritants, and bone involvement considered important contributing factors. Consequently, long‐term clinical follow‐up is recommended, particularly in pediatric patients, in whom preservation of developing dentition and craniofacial growth are essential considerations. [1, 3, 5, 14, 15]

Despite the large number of reports describing PGCG, cases demonstrating radiographic cortical erosion severe enough to resemble CGCG remain exceptionally uncommon, particularly in children. Consequently, the available literature provides limited guidance regarding the diagnostic sequence required to differentiate these entities and avoid overtreatment. This diagnostic challenge becomes even more relevant when the lesion exhibits clinical, radiographic, and histopathological findings that overlap with aggressive intraosseous giant cell lesions.

In the present report, we describe an unusual pediatric case of PGCG presenting with cortical bone erosion and imaging findings highly suggestive of CGCG. The final diagnosis was established only after sequential clinical evaluation, incisional biopsy, cone‐beam computed tomography (CBCT), laboratory assessment to exclude metabolic bone disease, and definitive histopathological examination following complete surgical excision. This case highlights the importance of comprehensive clinicopathological and radiographic correlation when evaluating giant cell lesions of the jaws and discusses the current evidence regarding their differential diagnosis and management.

## 2. Case Presentation

A previously healthy 10‐year‐old boy was initially evaluated in the Emergency Department of the Instituto Nacional de Pediatría, Mexico City, Mexico, because of a rapidly enlarging gingival mass involving the right mandibular alveolar ridge. During the initial emergency assessment, both the patient and his father reported that the lesion had enlarged over the preceding 2 weeks, raising concern for an aggressive process and prompting urgent referral to the Department of Pediatric Stomatology for further evaluation.

During the pediatric stomatology consultation, a comprehensive history was obtained. Upon further questioning, both the patient and his father recalled that a small gingival swelling had first appeared approximately 8 months before presentation. According to their account, the lesion had remained relatively stable for several months before undergoing a marked increase in size during the 2 weeks preceding consultation. The patient denied pain, spontaneous bleeding, fever, weight loss, or any other systemic symptoms. His medical history was otherwise unremarkable, with no history of facial trauma, systemic disease, or medication use.

**Figure 1:**
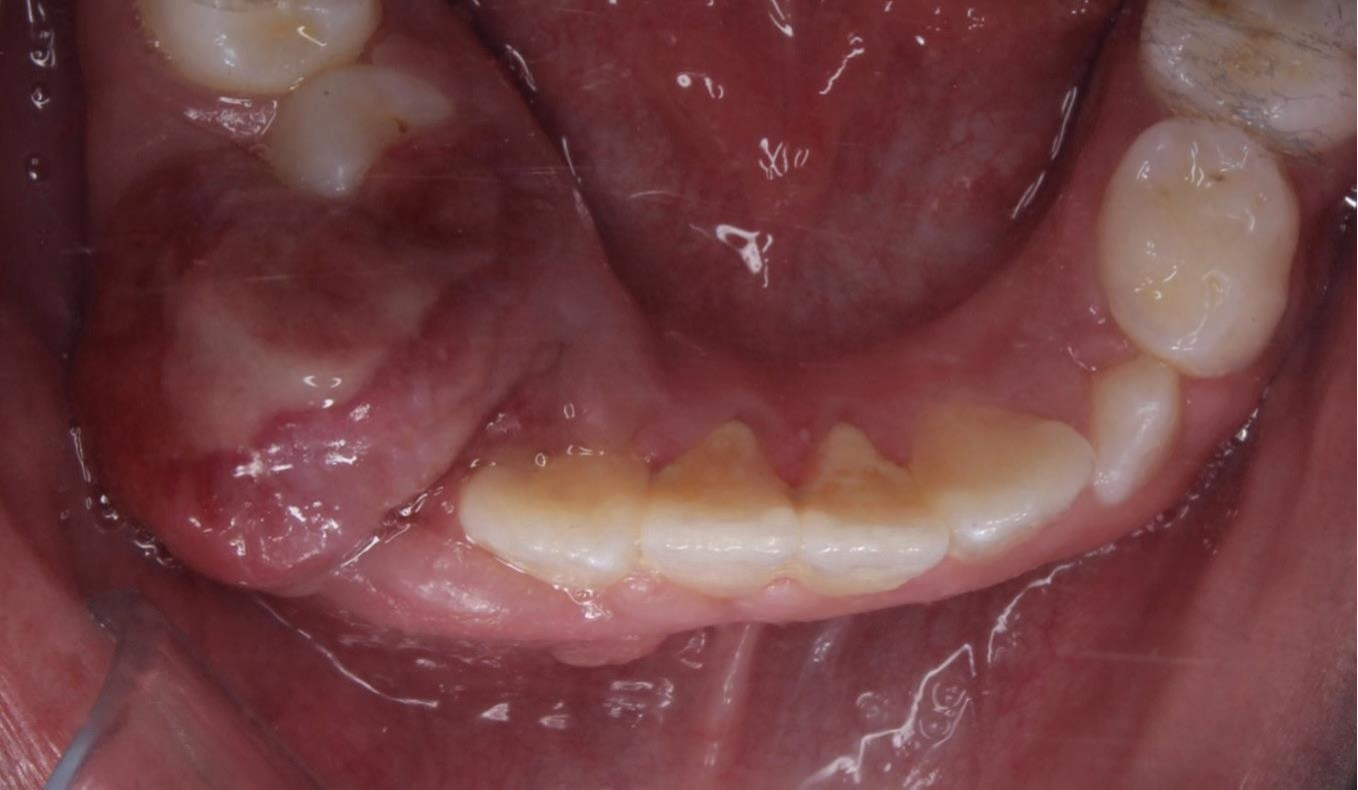
Clinical presentation of the lesion at the initial consultation. A large sessile exophytic gingival mass involving the right mandibular alveolar ridge extending from the primary right canine to the first premolar. The lesion exhibited an erythematous‐to‐violaceous appearance with focal lobulation.

Extraoral examination revealed no facial asymmetry or palpable cervical lymphadenopathy. Intraoral examination demonstrated late mixed dentition and a solitary sessile exophytic gingival mass measuring approximately 4 × 3 cm, located on the buccal gingiva of the right mandibular alveolar ridge and clinically extending from the primary right canine to the first premolar (Figure 1). The lesion exhibited a smooth, focally lobulated surface with an erythematous‐to‐violaceous coloration and a firm consistency on palpation. It was asymptomatic; however, repeated trauma during occlusion caused intermittent inflammation because the lesion came into contact with the opposing maxillary teeth. Oral hygiene was poor, and multiple untreated carious lesions were also observed.

**Figure 2:**
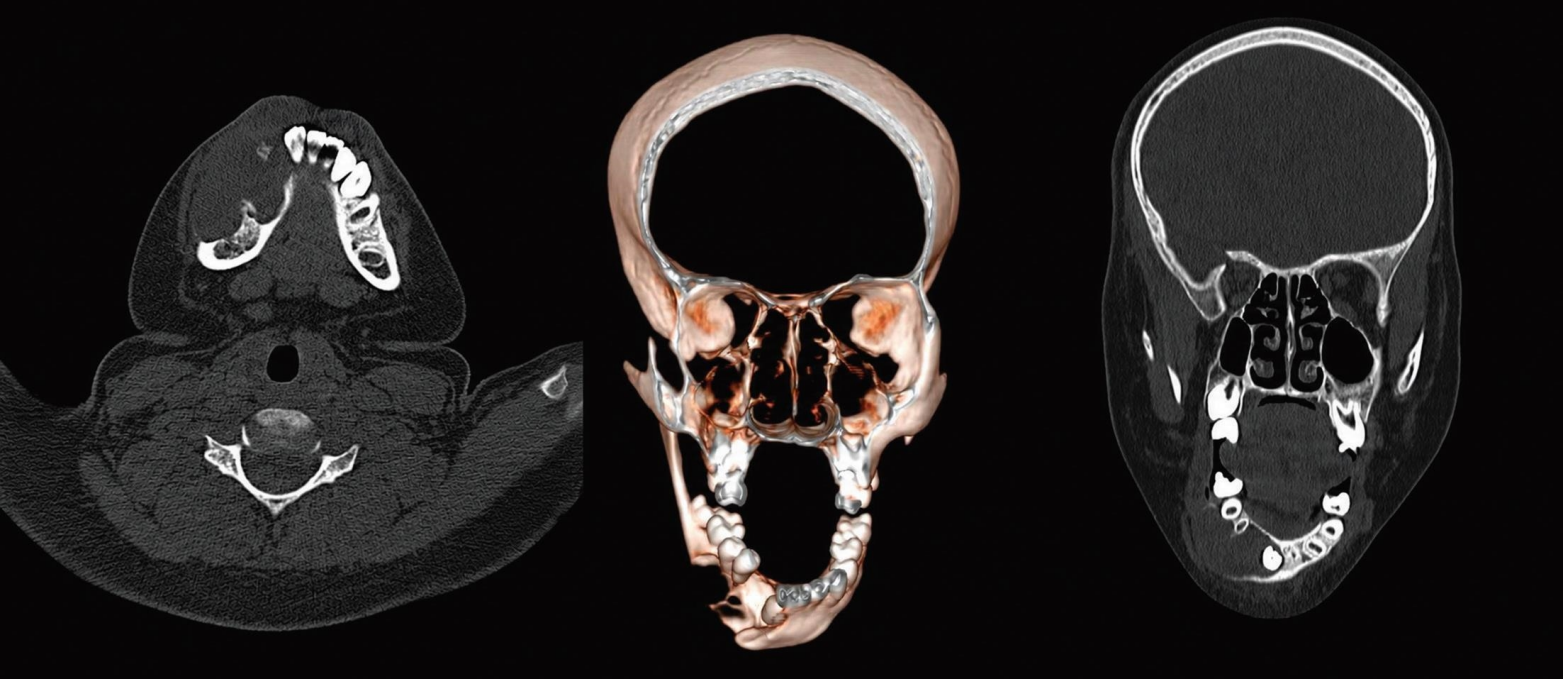
Cone‐beam computed tomography demonstrating a 40 × 25 mm expansile osteolytic lesion involving the right mandibular body with cortical bone erosion, involvement of the adjacent premolars, and surrounding the crown of the impacted permanent canine. Arrows indicate the areas of cortical destruction.

**Figure 3:**
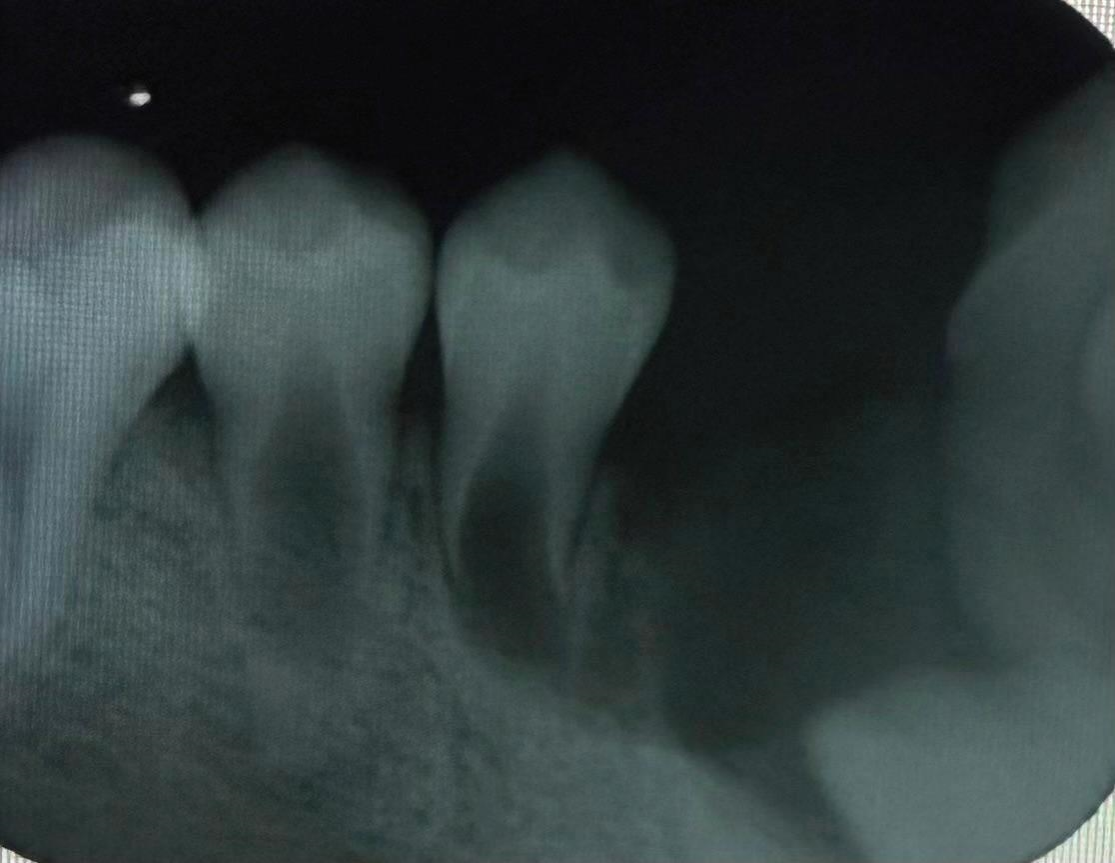
Intraoral periapical radiograph illustrating the extent of cortical involvement of the right mandibular body.

Initial panoramic and periapical radiographs demonstrated cortical alteration involving the right mandibular body. Because the degree of apparent osseous involvement was disproportionate to the clinical appearance of a peripheral gingival lesion, CBCT was obtained to determine the true extent of the lesion and whether it originated within the underlying bone. CBCT demonstrated a 40 × 25 mm expansile osteolytic lesion (estimated volume, 13 cm^3^) involving the right mandibular body, with partially defined margins and heterogeneous internal density, predominantly solid with focal hypodense areas (Figure 2). The lesion produced cortical bone erosion, apparently caused resorption of the primary right canine, involved the roots of both premolars, and surrounded the crown of the ipsilateral impacted permanent canine. Although the soft tissue lesion appeared clinically confined to the gingiva, CBCT demonstrated more extensive involvement of the underlying alveolar bone and adjacent developing teeth. These findings suggested a locally aggressive intraosseous process, and the principal radiographic differential diagnoses included CGCG and odontogenic myxoma, despite the clinically peripheral appearance of the lesion (Figure 3).

**Figure 4:**
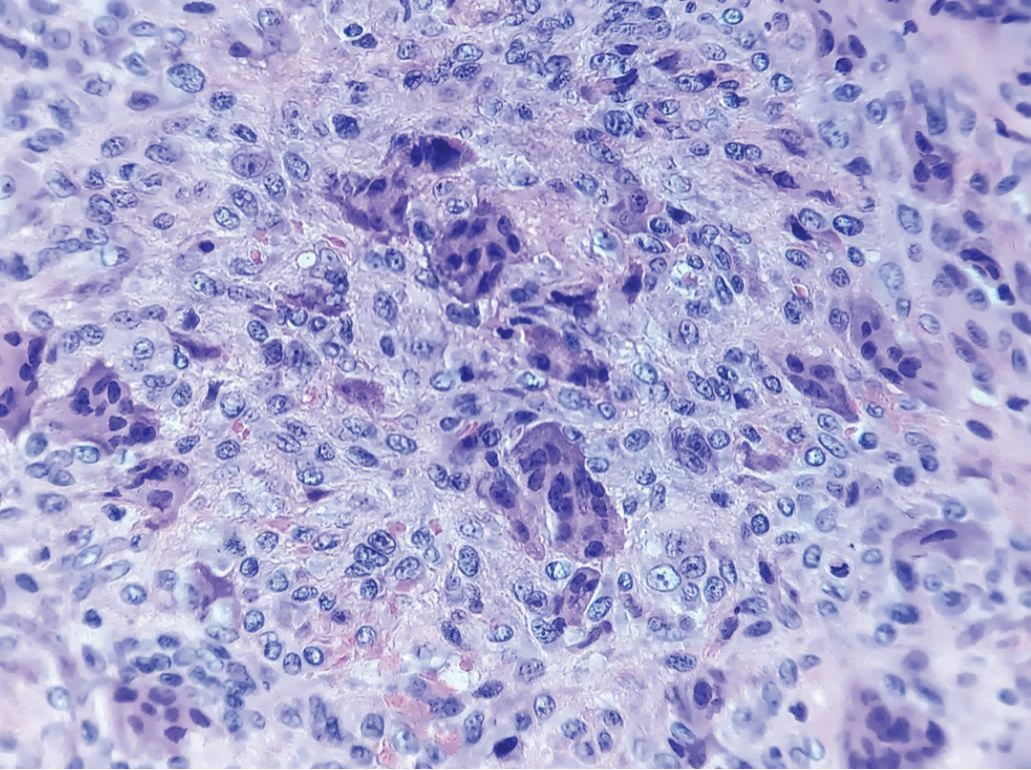
Histopathological examination (hematoxylin and eosin stain) demonstrating numerous multinucleated giant cells distributed within a fibrovascular stroma containing spindle‐shaped mononuclear cells, areas of hemorrhage, and hemosiderin deposition, consistent with peripheral giant cell granuloma.

To establish a tissue diagnosis before definitive treatment, an incisional biopsy was performed under local anesthesia. Histopathological examination revealed numerous multinucleated giant cells dispersed within a highly cellular fibrovascular stroma containing areas of hemorrhage and hemosiderin deposition, findings consistent with PGCG (Figure 4). However, because these microscopic features overlap considerably with those of CGCG and Brown tumor associated with hyperparathyroidism, histopathology alone was insufficient to establish a definitive diagnosis.

Therefore, laboratory investigations, including serum calcium, phosphorus, alkaline phosphatase, and parathyroid hormone levels, were requested to exclude an underlying metabolic bone disorder. All biochemical parameters were within normal limits, effectively ruling out hyperparathyroidism. Integration of the patient′s clinical history, intraoral findings, radiographic characteristics, laboratory investigations, and histopathological features established the diagnosis of PGCG despite imaging features that strongly suggested a central giant cell lesion.

**Figure 5:**
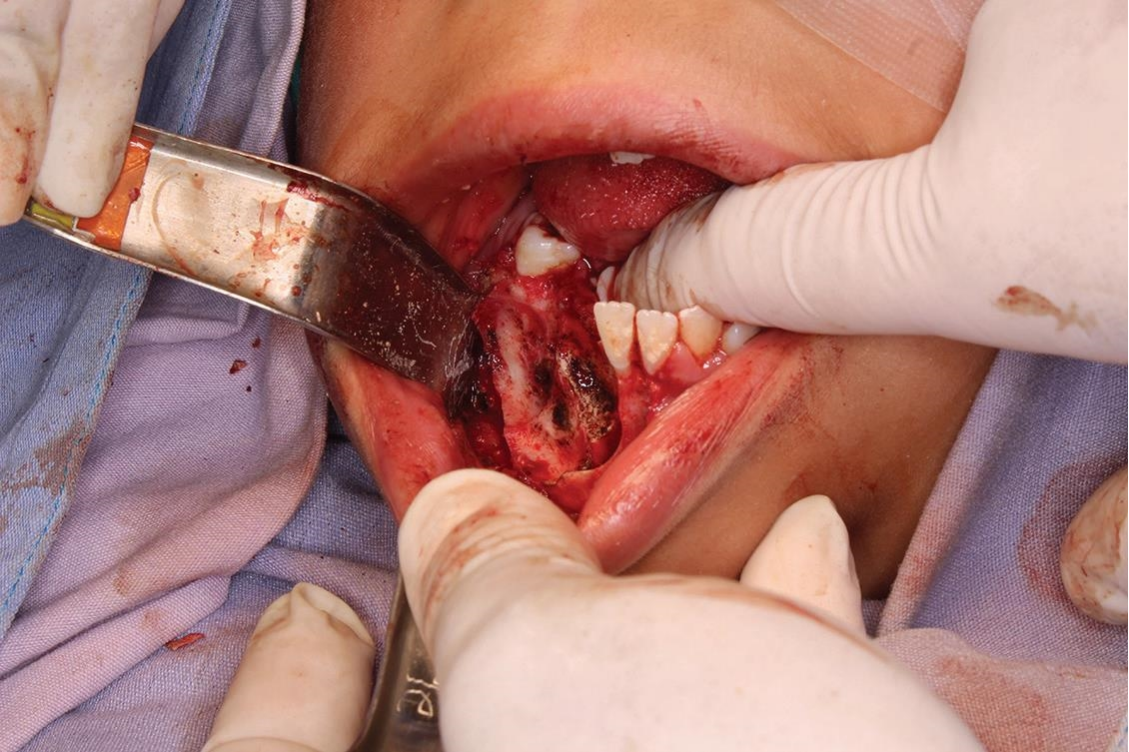
Intraoperative photographs demonstrating complete surgical excision of the lesion, peripheral osteotomy, and immediate postoperative appearance.

Considering the patient′s age, the lesion size, and the anticipated surgical complexity, complete surgical excision was performed under general anesthesia. Following local infiltration with 2% lidocaine containing 1:100,000 epinephrine to improve hemostasis, the lesion was excised using electrosurgery with blunt dissection extending to the periosteum. Peripheral osteotomy of the underlying alveolar bone was subsequently performed to reduce the risk of recurrence. Hemostasis was achieved using oxidized regenerated cellulose (Surgicel), and the surgical wound was closed with 4‐0 polyglactin (Vicryl) sutures (Figure 5).

Histopathological examination of the excisional specimen confirmed the diagnosis of PGCG, with microscopic findings consistent with those observed in the incisional biopsy.

At the time of manuscript preparation, the patient had not yet attended the scheduled postoperative follow‐up appointment. Consequently, clinical examination and follow‐up imaging could not be performed. The patient′s family has been contacted, and continued clinical and radiographic surveillance has been recommended given the possibility of recurrence reported for giant cell lesions.

## 3. Discussion

PGCG is a benign reactive lesion arising from the periosteum or periodontal ligament in response to chronic local irritation or trauma. Although it is among the most common peripheral giant cell lesions affecting the oral cavity, its occurrence in pediatric patients remains uncommon. Clinically, PGCG usually presents as a slowly growing exophytic gingival mass without significant involvement of the underlying bone. The present case is unusual because the lesion demonstrated rapid enlargement, cortical bone erosion, and radiographic features highly suggestive of CGCG, creating a considerable diagnostic challenge. This case highlights that, despite its peripheral origin, PGCG may occasionally exhibit aggressive radiographic characteristics that mimic central giant cell lesions, emphasizing the importance of integrating clinical examination, imaging, histopathological findings, and laboratory investigations before establishing a definitive diagnosis [1–3, 6, 9].

PGCG accounts for approximately 5%–10% of benign oral lesions and most frequently affects adults during the 4th–6th decades of life, with a slight female predominance. Pediatric cases are considerably less common and generally present as reddish‐blue sessile or pedunculated gingival masses involving the mandibular gingiva anterior to the molars. Chronic irritation from dental plaque, calculus, defective restorations, erupting teeth, trauma, or orthodontic appliances has been proposed as the principal etiologic stimulus, although the precise pathogenic mechanisms remain incompletely understood. In the present patient, poor oral hygiene, multiple untreated carious lesions, and repeated traumatic contact with the opposing dentition were considered potential contributing factors. However, these local irritants alone do not adequately explain the extensive cortical erosion observed radiographically, suggesting that certain PGCGs may demonstrate more aggressive biological behavior than is traditionally expected [1–3, 6, 7, 9, 14].

One of the most remarkable aspects of this case was the discrepancy between the clinical presentation and the radiographic findings. Clinically, the lesion appeared confined to the gingival soft tissues, favoring a diagnosis of PGCG. Conversely, CBCT revealed an expansile osteolytic lesion associated with cortical bone erosion, involvement of adjacent developing teeth, and extension around the impacted permanent canine, findings far more characteristic of CGCG or other intraosseous lesions, such as odontogenic myxoma. Although superficial “cupping” resorption of the alveolar crest has occasionally been reported in PGCG, extensive cortical erosion remains distinctly uncommon, particularly in children. Similar findings have only been described sporadically in isolated case reports and small case series, suggesting that long‐standing peripheral lesions may induce pressure‐related remodeling or superficial cortical destruction sufficient to mimic aggressive central lesions. Consequently, advanced imaging alone may not reliably differentiate PGCG from CGCG when atypical radiographic features are present [3, 5–7, 9, 15].

The principal diagnostic dilemma in this patient involved distinguishing PGCG from CGCG. Both lesions share numerous histopathological features, including multinucleated giant cells distributed within a fibrovascular stroma containing areas of hemorrhage and hemosiderin deposition, making microscopic examination alone insufficient for a definitive diagnosis. However, the biological behavior and therapeutic implications of these lesions differ substantially. CGCG is an intraosseous lesion capable of cortical perforation, root resorption, tooth displacement, and local recurrence, frequently requiring more extensive surgical procedures or adjunctive pharmacologic therapies. In contrast, PGCG is a reactive soft tissue lesion generally managed by complete local excision combined with elimination of local irritants. Therefore, definitive diagnosis requires careful correlation of clinical findings, radiographic characteristics, intraoperative observations, and histopathological examination. In the present case, although CBCT strongly suggested a central giant cell lesion, the gingival origin identified clinically and confirmed during surgery, together with the histopathological findings, established the diagnosis of PGCG [2, 3, 8, 10–13].

Another important aspect of this case was the complementary role of laboratory investigations. Because multinucleated giant cell lesions of the jaws may also represent the oral manifestation of hyperparathyroidism (Brown tumor), biochemical evaluation is recommended whenever the clinical or histopathological findings are inconclusive. Brown tumors are histologically indistinguishable from PGCG and CGCG and therefore require exclusion through assessment of serum calcium, phosphorus, alkaline phosphatase, and parathyroid hormone levels. In the present case, all biochemical parameters were within normal limits, effectively excluding hyperparathyroidism and reinforcing the diagnosis of PGCG. This multidisciplinary diagnostic approach prevented inappropriate treatment and illustrates that histopathological findings should always be interpreted within the context of clinical, radiographic, and laboratory information [4, 8, 10–12].

The present case also demonstrates the diagnostic value of CBCT in evaluating atypical peripheral giant cell lesions. Conventional radiographs suggested osseous alteration but were insufficient to determine the true extent of the lesion or its relationship with adjacent anatomical structures. CBCT provided detailed three‐dimensional assessment of cortical integrity, adjacent tooth involvement, and the relationship between the lesion and the impacted permanent canine, information that significantly influenced the initial differential diagnosis and surgical planning. Nevertheless, despite its superior spatial resolution, CBCT alone could not differentiate a peripheral reactive lesion from a CGCG. This finding reinforces that advanced imaging should be considered an adjunctive diagnostic tool rather than a definitive method for distinguishing giant cell lesions of the jaws [3, 8, 10, 12].

Complete surgical excision remains the treatment of choice for PGCG and should include elimination of local irritating factors together with removal of the lesion down to the periosteum to reduce the likelihood of recurrence. Recurrence rates reported in the literature range from approximately 5% to 20% and are generally attributed to incomplete excision or persistence of local irritants. Considering the lesion size, cortical involvement, and proximity to developing permanent teeth, complete excision under general anesthesia was considered the safest therapeutic approach in the present patient. Peripheral osteotomy was additionally performed to remove potentially involved periosteal tissues and minimize recurrence risk. At the time of manuscript preparation, the patient remained free of clinical recurrence with satisfactory soft tissue healing, although long‐term radiographic surveillance remains necessary because postoperative imaging has not yet been completed [1, 3, 5–7, 9, 14].

This report has certain limitations. Postoperative radiographic follow‐up is not yet available because the patient resides outside Mexico City, making regular attendance at our institution difficult. Nevertheless, serial clinical evaluations have demonstrated satisfactory healing without evidence of recurrence, and radiographic follow‐up has already been scheduled as part of the patient′s long‐term surveillance. Despite this limitation, the present case contributes valuable evidence regarding an uncommon presentation of PGCG in childhood and emphasizes the importance of recognizing that reactive peripheral lesions may occasionally exhibit imaging characteristics suggestive of aggressive central pathology [1, 3].

This case emphasizes that PGCG should remain within the differential diagnosis of pediatric gingival lesions even when advanced imaging demonstrates cortical erosion suggestive of a CGCG. Accurate diagnosis depends on the integration of clinical examination, advanced imaging, histopathological evaluation, and biochemical investigations rather than reliance on a single diagnostic modality. Awareness of this uncommon presentation may facilitate earlier recognition, prevent misdiagnosis, and guide appropriate treatment while avoiding unnecessarily aggressive management of benign reactive lesions [1–3, 6, 8–12].

## 4. Conclusion

PGCG should be considered in the differential diagnosis of pediatric gingival lesions, even when advanced imaging demonstrates cortical bone erosion suggestive of a CGCG. This case highlights that atypical radiographic findings alone should not determine the final diagnosis. Instead, accurate diagnosis relies on the integration of clinical examination, CBCT, histopathological evaluation, and laboratory investigations to exclude systemic conditions with similar microscopic features. Early recognition of this uncommon presentation may prevent misdiagnosis, avoid unnecessarily aggressive treatment, and facilitate appropriate surgical management with favorable clinical outcomes.

## Author Contributions

**Dante Alejandro Pineda Noceda:** conceptualization, investigation, data curation, formal analysis, writing – original draft, writing – review and editing, visualization. **Raúl Mendoza García:** investigation, resources, surgical management, writing – review and editing. **Yamely Ruiz Vázquez:** validation, formal analysis, histopathological diagnosis, writing – review and editing. **María Elena Llarena del Rosario:** supervision, project administration, writing – review and editing.

## Funding

No funding was received for this manuscript.

## Disclosure

All authors read and approved the final version of the manuscript.

## Ethics Statement

Ethical approval was not required because this manuscript describes a single anonymized case report with written informed consent obtained from the patient′s legal guardian. All procedures were performed in accordance with the ethical standards of our institution and the Declaration of Helsinki.

## Consent

Written informed consent was obtained from the patient′s parent/legal guardian for publication of this case report and the accompanying clinical and radiographic images. A copy of the written consent is available for review by the editor‐in‐chief upon reasonable request.

## Conflicts of Interest

The authors declare no conflicts of interest.

## Data Availability

The data that support the findings of this study are available on request from the corresponding author. The data are not publicly available due to privacy or ethical restrictions.
